# High rates of aneuploidy, mosaicism and abnormal morphokinetic development in cases with low sperm concentration

**DOI:** 10.1007/s10815-019-01673-w

**Published:** 2020-01-04

**Authors:** Semra Kahraman, Yucel Sahin, Hakan Yelke, Yesim Kumtepe, Mehmet A. Tufekci, Cigdem C. Yapan, Mesut Yesil, Murat Cetinkaya

**Affiliations:** Assisted Reproductive Technologies and Reproductive Genetics Center, Istanbul Memorial Hospital, Piyalepasa Bulvari, Sisli, 34384 Istanbul, Turkey

**Keywords:** Severe male factor, PGT-A, Chromosomal status, Embryo morphokinetics

## Abstract

**Purpose:**

The aim of our study was to evaluate the impact of severe male infertility (SMF) on the chromosomal status of embryos and any possible correlation between chromosomal status and embryo morphokinetics in younger women using data obtained from comprehensive preimplantation genetic tests.

**Methods:**

The trial was conducted in an ART and Reproductive Genetics Centre between 2011 and 2018. A total of 326 cycles in cases with SMF where the female partner’s age was ≤ 35 years were evaluated. SMF is defined as sperm concentration below 5 mil/ml (million per milliliter) and divided into three subgroups according to sperm concentrations: 1–5 mil/ml, < 1mil/ml and testicular sperm. The control group of 190 cycles had normal sperm parameters.

**Results:**

Significantly lower chromosomal euploidy rates were found in the testicular sperm group compared with the normal sperm controls when the female age was ≤ 35 years. In SMF, statistically significantly affected chromosomes were 2, 10, 11, 17, 21 and sex chromosomes. The mosaicism and abnormal morphokinetic development rates were higher in the SMF group than in control group, and this difference was significant when testicular sperm was used.

**Conclusion:**

Lower euploidy rates, higher mosaicism rates and a higher incidence of abnormal morphokinetic development were observed in cases with testicular sperm with female partners ≤ 35 years compared with normal sperm controls. These findings suggest that PGT-A may be advisable in severe male infertility cases. Furthermore, the correlation between morphokinetics and chromosomal status was greatly reduced or absent in these most severe forms of male infertility, thus the need for new morphokinetic models.

**Electronic supplementary material:**

The online version of this article (10.1007/s10815-019-01673-w) contains supplementary material, which is available to authorized users.

## Introduction

Male infertility is a factor in approximately 50% of ART cases. Of these, just over 20% are diagnosed with severe male factor infertility, which is defined as sperm concentration below 5 million per ml [1]. Since male factor is one of the most commonly seen indications for ART, the relationship between severe male infertility and embryo aneuploidy has long been a subject of interest. However, most studies into this were based on data obtained using FISH [2–5]. There have been only a limited number of studies using comprehensive chromosomal analysis to investigate this relationship [6, 7]. Coates et al. (2015) reported that the use of suboptimal sperm increases the risk of sex chromosome abnormalities in preimplantation blastocyst embryos using aCGH (array comparative genomic hybridization) [6], whereas, in addition, in our center, in cases where NGS (next generation sequencing) was used, we were also able to evaluate mosaicism.

Mazzili et al. (2017), reporting on the effect of the male factor on the clinical outcome of intracytoplasmic sperm injection combined with preimplantation aneuploidy testing, concluded that the euploidy rate and implantation potential of the obtained blastocysts are independent from sperm quality but that SMF impairs early embryonic competence regarding fertilization rate and developmental potential [7]. Mazzili et al. investigated chromosomal abnormality in 54 embryo transfer (ET) cycles with young female partners (< 35 years) in SMF subgroups using qPCR [7], whereas our study at Istanbul Memorial Hospital used aCGH and NGS to investigate 326 cycles with young female partners (≤ 35 years) according to severe male infertility subgroups ranging from 5 million/ml to non-obstructive azoospermia (NOA).

In SMF cases, defective centrosome plays an important role in the embryo development. Centrosome is paternally inherited and responsible for the first mitotic divisions after fertilization. Comizzoli et al. (2006) showed a higher proportion of zygotes with short or absent sperm asters after ICSI with testicular spermatozoa compared with ejaculated spermatozoa that contained large sperm asters after ICSI [8]. The poor pattern of aster formation from the testicular centrosome was associated with delayed first cleavage, slower developmental rate and reduced formation of morulae and blastocyst. Remarkably, improvement was reported when testicular sperm centrosome was replaced by a centrosome from an ejaculated spermatozoon, which resulted in higher rates of embryo development comparable with data from ejaculated spermatozoa.

Also, the relationship between sperm DNA fragmentation and male infertility has been a subject of interest in different studies. Although scarce reports exist on the relation between severe male infertility and the chromosomal status of embryos studied with comprehensive preimplantation genetic tests, sperm DNA fragmentation has been long debated and used by some laboratories as a predictive test for the outcome of IVF cycles especially with SMF. However, no definitive relationship was described between the sperm DNA fragmentation index (DFI) subgroups and blastocyst euploidy rate or morphological grading [9, 10]. In another study, sperm function tests like hyaluronan-binding assay, DNA fragmentation and hyperactivity were evaluated and found to be predictive of the fertilization rate and embryo quality but only in IVF cycles and not in ICSI cycles [11]. In an earlier study, low blastocyst rates were reported in cases with higher DFI (≥ 30%) when compared with < 30% in both IVF and ICSI cycles [12].

The aim of our study was to evaluate the impact of severe male infertility on the chromosomal status of embryos and any possible correlation between chromosomal status and embryo morphokinetics in younger women using data obtained from comprehensive preimplantation genetic tests.

## Material and methods

In this study, using findings from work in our center at Istanbul Memorial Hospital over the past 8 years, the impact of severe male infertility on chromosomal status and any correlation between chromosomal status and embryo morphokinetics were evaluated.

Firstly, the chromosomal status of embryos in SMF cases using initially aCGH (2011–2016) and latterly NGS (2017–2018), which included reference to mosaicism, was evaluated in male factor subgroups. Secondly, morphokinetic evaluation of embryos with time lapse was performed, and then any possible correlation between chromosomal status and morphokinetics was evaluated. Thirdly, pregnancy outcomes in younger women (≤ 35) according to severe male infertility subgroups were evaluated.

Severe male factor (SMF) is defined as sperm concentration below 5 million per ml. However, this covers a very wide range from 5 million down to non-obstructive azoospermia (NOA), in which sperm production is severely impaired.

In our study, SMF cases were divided into the following 3 subgroups: 2 ejaculated sperm subgroups according to sperm concentrations, (1) 1–5 mil/ml and (2) < 1 mil/ml, and (3), the third group, testicular sperm. The latter group was comprised of cases with obstructive azoospermia (OA) and non-obstructive azoospermia (NOA), OA being defined as the absence of spermatozoa in the ejaculate despite normal spermatogenesis and NOA is defined as no sperm in the ejaculate due to failure of spermatogenesis, which is the most severe form of male infertility. The control group was comprised of males with normal sperm parameters (using WHO criteria of > 39 million and > 40% motile sperm in the ejaculate) [13].

A total of 279 couples with 326 cycles where the female partner was 35 or younger were evaluated. Of these, 128 cases with 137 cycles (42.0%) had < 1 mil/ml sperm concentration; 115 cases with 150 cycles (46.0%) had sperm concentration of 1–5 mil/ml; and, in 36 cases, (12 cases with OA and 24 cases with NOA) testicular sperm was used in 39 cycles (12.0%). The control group of 165 cases with 190 cycles had normal sperm parameters. Throughout the study, OA and NOA are regarded as one group (testicular sperm group) because no statistically significant differences were observed between them in rates of aneuploidy and mosaicism nor in morphokinetic development.

The average, minimum and maximum ages of the males are included in the Supplementary Table 1. The male age means (min: 22 years–max: 47 years) were 34.7 ± 4.7 (*n* = 128) (< 1 mil/ml sperm group); 33.9 ± 3.8 (*n* = 115) (1–5 mil/ml sperm group); 33.3 ± 4.2 (*n* = 36) (testicular sperm group); and 32.8 ± 4.8 (*n* = 165) (control group). No statistically significant differences were observed regarding male age between these sperm subgroups and the control group (Supplementary Table 1). The female age means were 31.1 ± 3.5, 31.1 ± 3.7, 31.3 ± 3.9 and 30.8 ± 3.7 (< 1 mil/ml, 1–5 mil/ml, testicular sperm and control group, respectively).

This retrospective study was approved by our institutional review board (Number: 2019/004). Patients were informed about the treatment and procedures and written informed consent was obtained from all patients before starting IVF treatment, embryo biopsy and genetic analysis and transfer procedures. Patients were informed about the possibility of misdiagnosis and the cancelation of embryo transfer in the absence of euploid embryos.

### Ovarian stimulation

The stimulation protocols have been outlined previously [14]. For ovarian stimulation, gonadotropin-releasing hormone (GnRH) analogue suppression (short or long), GnRH antagonist protocol and recombinant follicle stimulating hormone (rFSH) or a combination of rFSH and recombinant luteinizing hormone (rLH) (Luveris; Merck, Switzerland) or human menopausal gonadotropin (HMG) (HMG, Ferring, Switzerland) were used. Oocyte retrievals were carried out 36 h after the injection of 250 mcg recombinant hCG (Ovitrelle; Merck, Switzerland) by transvaginal ultrasound guidance.

### Sperm preparation techniques

In this study, three different sperm preparation techniques were used according to sperm concentration. Samples collected from cases with normal sperm parameters and with 5–15 mil/ml were treated with the pellet swim-up technique in Human Tubal Fluid (HTF) (Cooper Surgical, USA) supplemented with 5% Human Serum Albumin (HSA) (Cooper Surgical, USA). For samples with 1–5 mil/ml, density gradient centrifugation technique was preferred using COOK Spermient Media (Australia), which was diluted with HTF to obtain the 80 and 40% gradient solutions. For samples with a low sperm concentration (≤ 1 mil/ml) and for testicular sperm, the preparation was done by applying the mini gradient technique [15]. Approximately 3 to 4 h after oocyte retrieval, the cumulus cells were enzymatically removed (Hyarolunidase 80 IU/ml, Irvine Scientific, USA), and oocytes were transferred to culture media (Life Global®, Belgium). ICSI was applied to all sperm groups as it is the standard technique in our center.

### Embryo morphology scoring

Blastocysts were scored in terms of degree blastocoel expansion, morphological appearance of in inner cell mass and trophectoderm cells before vitrification according to Gardner’s classification (114–120 h post-ICSI) and classified into three groups: top quality (TQ), good quality (GQ) and poor quality (PQ) blastocysts [16]. The TQ designation includes 3AA, 4AA and 5AA blastocysts, whereas GQ comprises those graded as 3/ 4/ 5BB, AB or BA. Blastocysts of inferior quality were designated as PQ blastocysts.

### Embryo culture and incubation

Each of the 12 individual wells of the EmbryoSlide® (Vitrolife, Sweden) culture dish was filled with 25 μl of a single step culture medium (Life Global®, Belgium), supplemented with 10% Plasmanate (Life Global®, Belgium), and all wells were covered with an overlay of 1.5 ml paraffin oil (Life Global®, Belgium). Following ICSI, injected oocytes were positioned in the wells of the slide, which was placed in a time-lapse incubator (EmbryoScope™, Sweden) at 6% CO_2_, 5% O_2_ and 37 C with the adjustment of pH to 7.26–7.30 for 5 days until embryo transfer. The culture medium was refreshed on the afternoon of day 3 by replacing the incubated slide with a new pre-equilibrated slide prepared as described above. Image stacks were acquired at seven focal planes every 15 min, and data were continuously transferred to an external computer, EmbryoViewer® workstation (Vitrolife, Sweden). Embryo development was annotated by one investigator and cross-checked by two other assessors.

### Time-lapse evaluation and embryo scoring

Morphokinetic variables for all cleavage events up to the expanded blastocyst stage were annotated. All relevant events, PN appearance and fading, t2, t3, t4, t5, t6, t7, t8, t9, tM, tSB and tB, were recorded in the EmbryoViewer® workstation for the embryos reached that specific developmental stages. The time of all mitotic events was expressed as hours post-ICSI for the embryos reaching these developmental phases. In order to minimize the variation of ICSI time within the oocytes of each patient, ICSI was split between two embryologists above ten oocytes. Therefore, the maximum ICSI duration did not exceed 15 min, which is below the default time interval of each picture taken by the camera of the EmbryoScope™ system. tM was annotated at the end of the compaction process, when compaction was observed to be full with no apparent cell contours. tSB marks the initiation or start of blastulation, the first frame when the initiation of a cavity formation is observed. tB indicates a blastocyst, where the ICM and the cavity are formed. tEB shows an expanded blastocyst with 50% thinning of the zona pellucida. Only embryos reaching blastocyst on time, 114–120 h post-ICSI, were analyzed for the purposes of morphokinetics. Therefore, embryos that were either slow growing or arrested before reaching blastocyst were excluded from the morphokinetic evaluation.

Blastocysts were scored according to Gardner’s classification and selected for transfer based on the final morphology 114–120 h post-ICSI.

### Trophectoderm biopsy

Trophectoderm biopsy involved making a hole in the zona pellucida by using diode laser (RI Saturn 3, England) on day 3 of embryonic development, which allowed the developing trophectoderm cells to protrude after blastulation, facilitating the biopsy. On day 5 after fertilization, between five and eight cells were excised using laser energy, without loss of inner cell mass. If the embryo was on day 6 or the hatching part of the embryo had excessive trophectoderm cells, both laser and mechanic techniques were used. Mechanical cut is used by sliding biopsy pipette with 30-mm inner diameter (Origio, Denmark) down to holding pipette (Origio, Denmark). If small number of cells protruded or trophectoderm score was B, then detachment was done by mechanical cutting only.

### Preimplantation genetic testing (PGT-A)

In the scope of this study, two PGT-A techniques were used, aCGH (array comparative genomic hybridization) and NGS (next generation sequencing).

aCGH was performed between 2011 and 2016 using 24Sure kit (Illumina, USA) following standard procedures on the provided manual. Analyses were done using BlueFuse Multi Analysis Software (Illumina, USA), illustrating the chromosome copy numbers in a biopsy sample.

NGS was performed between 2017 and 2018 using ReproSeq kit (ThermoFisher, USA) and initially PGM (Ion Personal Genome Machine, ThermoFisher, USA) and latterly S5 (ThermoFisher, USA). Analyses were performed on Ion Reporter software suit v5.2 and v5.6 (ThermoFisher, USA).

Mosaicism was only determined in NGS-tested samples; the range of mosaicism was identified between 20–80% as stated in Preimplantation Genetic Diagnosis International Society (PGDIS) guidelines (http://pgdis.org/docs/newsletter_071816.html). The copy number detection resolution of both aCGH and NGS techniques is similar in terms of base pairs. However, as NGS is based on numerical counting of reads and not logarithmic comparison, it can identify mosaicism in biopsies. Beyond mosaicism, there is no difference between these techniques in aneuploidy detection. Mosaicism was reported only when NGS was performed.

### Embryo vitrification and thawing

Good or top-quality blastocysts (at least 3BB) were vitrified on day 5 and day 6 mornings with Kitazato vitrification media (Kitazato, Japan) according to the manufacturer’s instructions, using Cryotop® as carrier. Blastocysts were thawed with Kitazato warming media according to manufacturer’s instructions. Embryos were first checked 30 min after thawing for immediate survival. A second check occurred 2 h after warming for re-expansion, hatching, extensive cytoplasmic granulation and the presence of necrotic foci, which are predictors of the rates of implantation, pregnancy and live birth [17]. Eligible blastocysts with at least 80% re-expansion and vitality were transferred in the afternoon of the same day.

### Luteal phase support

For luteal phase support, patients received a twice daily dose of progesterone gel administered intravaginally (Crinone® 8%; Merck Serono, Switzerland). Nine days after blastocyst transfer, serum β-hCG was measured. When pregnancy occurred, progesterone was continued until the 12th week of gestation. At 7 weeks, a transvaginal ultrasound was performed to monitor early pregnancy. A viable pregnancy was defined as the presence of fetal heartbeat and ongoing pregnancy was defined as a 12-week viable pregnancy.

### Statistics

Statistical analyses were done in MedCalc Statistical Software version 18.11.6 (MedCalc Software bvba, Belgium). For categorical comparisons, Chi-Square Test was used. For continuous data, Mann–Whitney U Test was used as the distribution of the tested variables was not normally distributed. Kruskal–Wallis test was used to analyze the effect of a classification factor with more than two groups on continuous variables. The threshold for statistical significance was taken as a *p* value of 0.05.

## Results

**Fig. 1:**
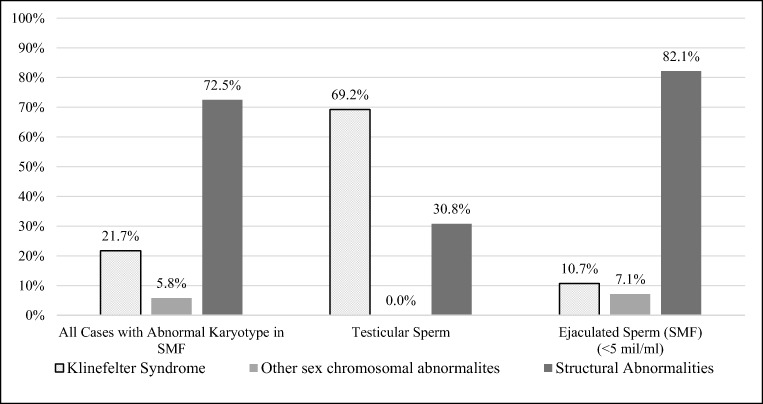
Distribution of karyotype abnormalities in all cases with abnormal karyotype in SMF and according to the sperm source used between 2011 and 2018 (*n* = 279)

Figure 1 shows the distribution of karyotype abnormalities in all cases with abnormal karyotype in SMF and according to the sperm source used between 2011 and 2018 (*n* = 279). The total percentage of cases with abnormal karyotype was 24.7%, which is significantly higher than the 4.2% found in the normal population [18]. Of these SMF cases, 5.4% presented Klinefelter’s Syndrome (47XXY), 1.4% other sex chromosomal abnormalities such as 47XYY and 17.9% structural chromosomal abnormality. In the testicular sperm group the percentage of chromosomal abnormalities was strikingly elevated at 36.1%. Of these, the vast majority (34.6%) were Klinefelter’s syndrome and 11.1% had structural rearrangement, whereas, in the ejaculated sperm group, the most frequent abnormality was translocations (18.9%). 2.4% had Klinefelter’s Syndrome and 1.6% had other sex chromosomal abnormalities.

Our overall peripheric karyotype analysis results of 3276 (between 2003 and 2018) severe male infertility cases with less than 5 mil/ml sperm or with testicular sperm are shown in Supplementary Fig. 1**.**

When patients and cycle characteristics were evaluated in SMF cases with young female partners, no statistically significant difference was observed in mean female age, male age, BMI and AMH levels, nor in the mean number of cumulus oocyte complexes, nor in the mean number of MII oocytes obtained (Supplementary Table 1)**.** The only significant difference was the lower rate of fertilization in cases of testicular sperm compared with the control group, 77.3% vs 83%, respectively (chi-square test, *p* = 0.0019).

A total of 741 blastocysts were biopsied in 326 cycles and tested for PGT-A in study group (female age ≤ 35) throughout the study duration.

**Fig. 2:**
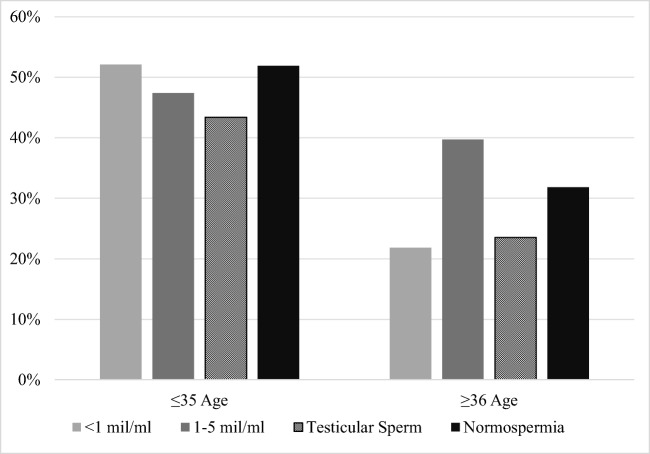
Euploidy rates in SMF groups according to female age (Chi-square, *p* < 0.001, *p* = 0.0001, respectively) (For the ≤ 35 age group: 282; 291; 168; 829 biopsies, for < 1 mil/1–5 mil/ml; testicular sperm; normospermia cycles, respectively) (For the ≥ 36 age group: 271; 234; 123; 1678, for < 1 mil/ml; 1–5 mil/ml; testicular sperm; normospermia cycles, respectively)

Figure 2 compares the overall chromosomal euploidy in SMF cases with young female partners (≤ 35) and with older female partners (≥ 36). The numbers of cycles and embryos in each subgroup with young female partners (≤ 35 years) were as follows: < 1 m/ml 150 cycles with 282 blastocysts; 1–5 m/ml, 137 cycles with 291 blastocysts; testicular sperm, 39 cycles with 168 blastocysts; and control group with normal sperm parameters, 190 cycles with 829 blastocysts. In order to investigate the effect of female age on chromosomal abnormality in SMF, subgroups and control group cases with older female partners (≥ 36) were also evaluated. The number of blastocysts in each subgroup with older female partners were as follows: < 1 mil/ml, 271 blastocysts; 1–5 mil/ml, 234 blastocysts; testicular sperm, 123 blastocysts; and normospermia, 1678 blastocysts.

Chromosomal euploidy rates were observed to be lowest in the testicular sperm cases when compared with other subgroups of SMF, and euploidy rates were statistically significantly lower than in the control group (*p* < 0.01). With the older female group (≥ 36) the chromosomal euploidy rates in SMF patients with testicular sperm and < 1mil/ml sperm were statistically significantly lower than in the 1–5 mil/ml and normospermia groups (*p* = 0.001).

A breakdown of aneuploidy results according to the number of chromosomes involved and segmental aneuploidy were evaluated (Table 1)**.** What is notable here is the significantly higher incidence of complex aneuploidy in testicular sperm cases (*p* = 0.004) and the relatively lower incidence of segmental aneuploidy in SMF groups.

**Table 1:** Chromosomal status of biopsied blastocysts according to SMF subgroups and control groups (741 embryos in SMF groups and 829 embryos in controls)

	< 1 mil/ml n:282 (%)	1–5 mil/ml n:291 (%)	Testicular sperm n:168 (%)	Normal sperm n:829 (%)
Euploid	52.1	47.4	43.4	51.9
Single Chromosome Aneuploidy	17.0	18.1	14.2	16.2
Double Chromosome Aneuploidy	5.7	8.2	5.3	6.3
Segmental Aneuploidy	6.7	7.5	4.8	8.8
Complex Aneuploidy	8.5	7.2	15.5	8.3
Mosaic *	10.9	15.6	22.0	9.9

*Mosaicism was diagnosed only in embryos analyzed with NGS

**Fig. 3:**
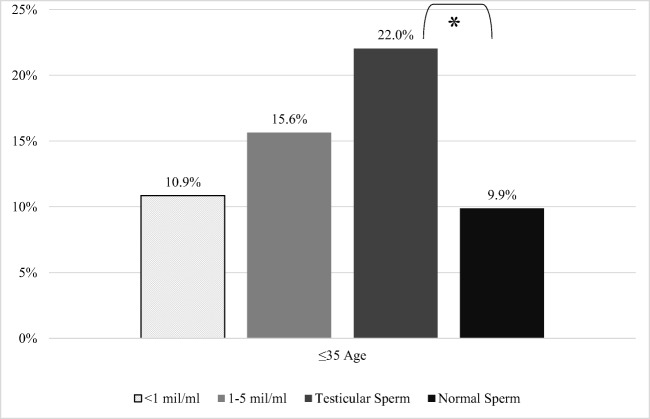
Mosaicism rates for NGS tested blastocysts in SMF groups (*n* = 596: 258; 211; 127 for < 1 mil/ml; 1–5 mil/ml and testicular sperm subgroups, respectively) and control group (*n* = 718), female age ≤ 35 (* Chi-Square test, *p* = 0.0001)

Mosaicism evaluation was performed, *n* = 596 embryos in SMF subgroups (< 1 mil/ml; 258 blastocysts, 1–5 mil/ml; 211 blastocysts and for azoospermia 127 blastocyts) and 718 blastocysts in control group) (Fig. 3). The highest mosaicism rate (22.0%) was observed when testicular sperm was used, and it was significantly higher than in cases with normal sperm parameters (9.9%) (*p* = 0.0001). In severe cases with less than 1 mil/ml sperm in the ejaculate, the mosaicism rate was lower but still higher at 10.9% than in the control group. In patients with 1–5 mil/ml, the rate was again a little lower but still high at 15.6%.

When the details of mosaicism were evaluated, overall mosaicism, double chromosomal mosaicism and complex mosaicism were significantly higher in the testicular sperm group when compared with the normozoospermia group (*p* = 0.0001, *p* = 0.0019 and *p* = 0.0033, respectively) (Table 2). Segmental mosaicism was also found in some cases; however, the numbers were very low that they were excluded from these statistics.

**Table 2:** Distribution of mosaic embryos analyzed with NGS

	< 1 mil/ml n:258 (%)	1–5 mil/ml n:211 (%)	Testicular sperm n:127 (%)	Normal sperm n:718 (%)
Mosaic embryos	10.9	15.6	22.0	9.9
Single Chromosomal Mosaicism	8.1	8.5	9.4	6.3
Double Chromosomal Mosaicism	1.2	4.7	7.9	2.5
Complex Mosaicism	1.6	2.4	4.7	1.1

The distribution of autosomal and sex chromosome abnormalities according to SMF, women age ≤ 35 all the way from chromosome 1 up to 22 and sex chromosomes for 1570 PGT-A blastocysts (741 for the study group, 829 for the control group) is shown in Supplementary Fig. 2**.** The most commonly observed autosomal chromosomal abnormalities in SMF cases were chromosomes 16, 21 and 22 followed by 2, 10, 11, 17 and sex chromosomes.

**Fig. 4:**
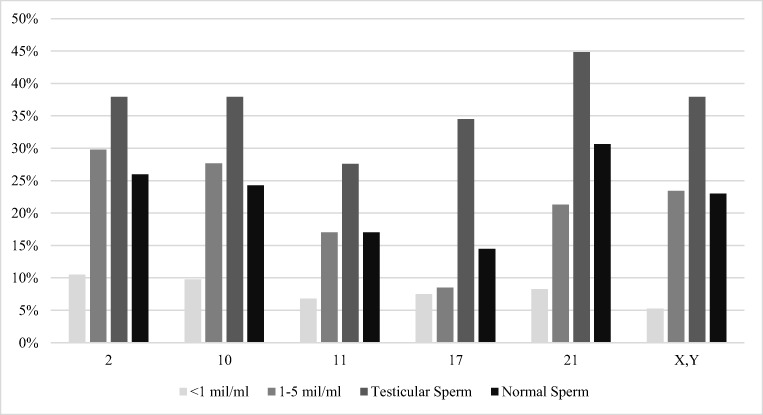
Distribution of 2, 10, 11, 17, 21 and sex chromosome abnormalities in SMF and normozoospermia group. (282; 291; 168; 829, for < 1 mil/ml; 1–5 mil/ml; testicular sperm and normospermia cycles, respectively)

As shown in Fig. 4, significantly different rates of abnormalities were observed for chromosomes 2,10,11, 17 and 21 and sex chromosomes in SMF groups with young female partners when compared with the control group (< 1 mil/ml, 282; 1–5 mil/ml, 291; testicular group,168; control group with normal sperm, 829 blastocysts. (Chi-Square Test; *p* = 0.0005, *p* = 0.005, *p* = 0.0082, *p* = 0.0009, *p* < 0.0001, *p* < 0.0001, respectively). For all the chromosomes tested, testicular sperm group had the highest aneuploidy rates when compared with the other SMF groups and control group.

The clinical outcomes of SMF groups are presented in Table 3**.** Once a chromosomally normal embryo was found for transfer, no significant difference was observed in clinical outcomes between the control group and the SMF groups. Although the clinical miscarriage rate was higher in the testicular sperm groups, this was not a significantly higher miscarriage rate.

**Table 3:** Clinical outcomes of PGT-A embryo transfer cycles in SMF cases with younger female partner (≤ 35 years)

	< 1 mil/ml (%)	1–5 mil/ml (%)	Testicular sperm (%)	Control group (%)	p*
BHCG +/ ET	68.6	68.9	74.2	71.3	0.837
Clinical Pregnancy	65.5	61.8	71.21	63.9	0.573
Clinical Miscarriage	15.5	12.1	23.4	12.2	0.347
Ongoing Pregnancy	53.4	54.3	54.5	52.9	0.897
Live Birth	52.9	52.7	53.1	51.3	0.916

*Chi-Square Test

The morphokinetic evaluation of 206 embryos in SMF groups and 421 embryos in the control group with young female partners (≤ 35) incubated in time-lapse incubator is shown in Supplementary Table 2 (< 1 mil/ml; 81 blastocysts, 1–5 mil/ml; 82 blastocysts, testicular group; 43 blastocysts; and control group;421 blastocysts were evaluated).

The group that had the statistically significantly greatest time difference in cleavage timings when compared with the normospermia group was the testicular group. Only embryos reaching blastocyst (114–120 post ICSI) during time-lapse culture were analyzed for the purposes of morphokinetics. Therefore, embryos that were either slow growing or arrested before reaching blastocyst were excluded from the morphokinetic evaluation. A significantly higher percentage of direct uneven cleavage was observed in the testicular sperm group when compared with the control group (27.7%, vs 20.7%, respectively, *p* < 0.0001). Also, significantly higher percentage of embryos were either slow growing or arrested (mostly at the cleavage stage) before reaching blastocyst (*p* < 0.001) was observed; 62.8% in the testicular sperm group, 47.4% in the < 1 mil/ml group, 47.3% in the 1–5 mil/ml group and 42.7% in the normozoospermia group, compared with normally fertilized oocytes.

In the testicular group, those which reached blastocyst stage did so significantly faster compared with all other groups. Indeed, the embryos in the testicular sperm group reached time-points from the first cleavage to the blastocyst stage faster than all the other groups; however, this difference was statistically significant for all time points only when compared with the normozoospermia group (Supplementary Table 2).

**Fig. 5:**
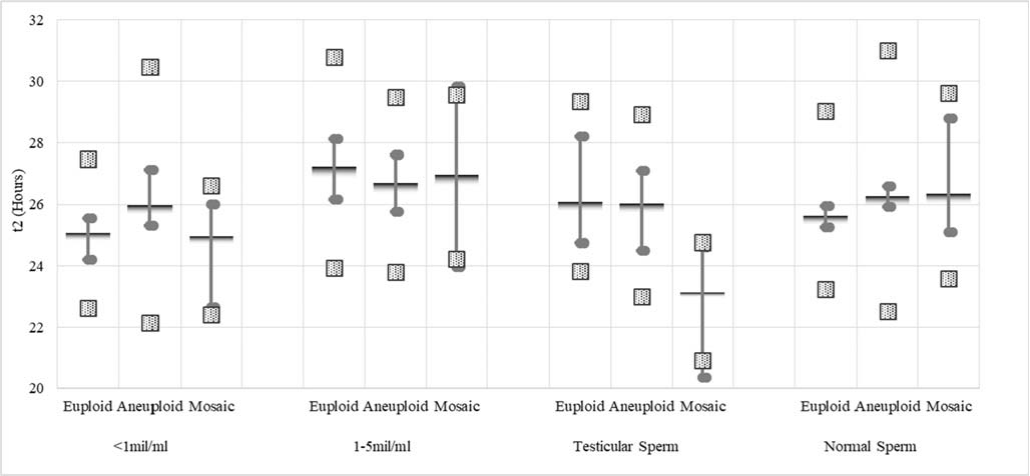
Embryo chromosomal status and morphokinetic development, time to first cleavage, in SMF subgroups (median values are depicted as horizontal lines, 95% CI for median are given as vertical lines and full circles, standard deviations as dotted squares)

When time to first cleavage was evaluated, in cases with normal sperm parameters, euploid embryos developed faster than aneuploid embryos (Fig. 5). However, when times to reach the 2-cell stage were compared, the cleavage timings were not statistically different for euploid and aneuploid embryos in the testicular sperm group.

**Fig. 6:**
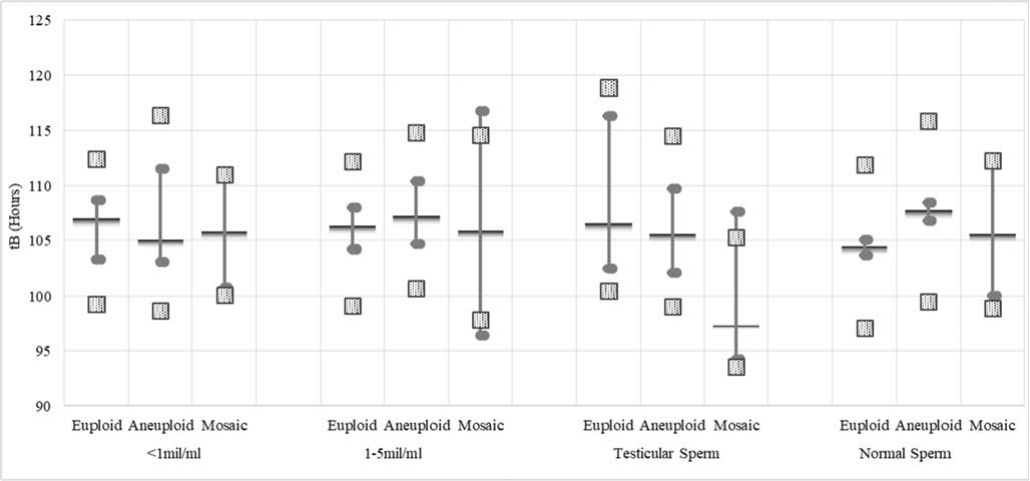
Embryo aneuploidy and morphokinetic development up to blastocyst stage in SMF groups (Median values are depicted as horizontal lines, 95% CI for median are given as vertical lines and full circles, standard deviations as dotted squares)

In the most severe forms, < 1 mil/ml and testicular sperm group, for tB timing, there was no statistically significant difference between euploid and aneuploid embryos (Fig. 6).

## Discussion

Using data obtained from comprehensive preimplantation genetic tests, the impact of severe male infertility on the chromosomal status of blastocysts was investigated. In order to exclude female age factor, SMF cases with young female partners, ≤ 35 were evaluated. Furthermore, a possible correlation between chromosomal status and embryo morphokinetics was evaluated.

The relationship between severe male infertility and embryo aneuploidy has long been a subject of interest*.* Our study differs from the two previous studies that focused on this area in three key aspects: Mazzili et al. investigated chromosomal abnormality in 1219 cycles but only 53 cycles with young female partners (< 35) using qPCR, whereas our study at Istanbul Memorial Hospital used aCGH and NGS to investigate 326 oocyte pick-up cycles with young female partners (≤ 35) [7]. In addition, our study evaluated mosaicism. Furthermore, embryo morphokinetics and any correlation with chromosomal status were investigated from the first cleavage to blastocyst stage.

As expected, between the SMF subgroups, the fertilization rate was found to be significantly lower than in the normal sperm group when testicular sperm were used. Similarly, Mazzili et al. (2017) found that the fertilization rate was significantly lower when testicular sperm, which are less mature and therefore less competent than ejaculated sperm [7]. Standard laboratory procedures were followed as clinically appropriate for each sperm concentration group in order to harvest viable sperm for ICSI from each group.

When chromosomal status was evaluated in blastocyst stage embryos in SMF subgroups with younger female partners, lowest chromosomal euploidy rates were found in the testicular sperm group. This difference was significant in the testicular sperm group where the rate was 43.4% compared with the approximate 65–70%, we would expect in younger female cases with male partners with normal sperm parameters [19, 20]. According to our data, peripheric karyotype abnormality was strikingly high at 36.1% in the testicular sperm group and 23.0% in the less than 5 mil/ml ejaculated sperm group. There may therefore be a correlation between high karyotype abnormality and chromosomal abnormality of embryos in the testicular sperm group.

As well as investigating SMF in cases with younger female partners, we also considered the effect of AMA (≥ 36 years) on embryo chromosomal status when combined with SMF. As expected, overall, the chromosomal abnormality rate in the older females’ group with SMF partners was higher than in SMF groups with younger female partners. Also, chromosomal abnormality rates between younger and older female partner groups in cases using testicular sperm was statistically significant (*p* < 0.0001). In addition to that, highest ratio of embryo aneuploidy was observed in testicular sperm group for younger patients. This finding may be related to evidence showing that a compromised testicular endocrine environment could increase the incidence of meiotic errors of spermatozoa [21]. Furthermore, immunocytogenetic studies of human spermatogenesis have revealed lower frequencies of meiotic recombination in NOA patients, suggesting a possible link to aneuploid sperm production [22]. There is therefore a clear need for further investigation into the incidences of chromosomal recombination and chromosomal breakage in severe male infertility cases. Munne et al. (2006) reported that sperm centrosomal disfunction contributes to embryonic aneuploidy, polyploidy and mosaicism [23]. Centrosomal defects can cause embryonic arrest through the formation of abnormal spindles and the accumulation of chromosomally abnormal cells that derive from them [24]. They reported molecular cytogenetic analysis of nine chromosomes in embryos from a patient with oligoastenozoospermia and slow or arrested cleavage-stage development on day 4 revealed a pattern of polyploidy and post-zygotic malsegregation of chromosomes that could be explained by an abnormal centrosomal distribution following cytokinetic failure and a defective spindle. All microtubule formations throughout development depend on sperm derived centriolar integrity; a defective centriol-centrosome complex inherited by a human oocyte may lead to abnormal chromosome separation with subsequent genomic instability, therefore compromising embryonic development [25, 26].

As far as we know, our study is the first to evaluate the mosaicism rate in SMF cases with young female partners. The mosaicism rate was higher in the severe male factor group than in the normal sperm group, and this difference was significant when testicular sperm was used. The mosaicism rate in embryos increased with the severity of male infertility. A significantly higher mosaicism rate was observed in cases where testicular sperm was used (*p* = 0.0001). It is known that ejaculated and testicular sperm differ in the degree of nuclear maturation. During spermiogenesis, the transit of spermatozoa in the epididymal tract favors DNA packaging by stabilizing the chromatine structure through protamine dephosphorylation and the formation of intra and intermolecular disulphide bridges between protamines as reported by [27]. This could suggest a possible link between testicular sperm and mosaicism.

In our study, the most commonly affected and statistically significant chromosomes identified in SMF cases were 2, 10, 11, 16, 17, 21 and sex chromosomes. The chromosomal abnormality rate in our overall PGT cases is 55% (mean female age 36.8 years) with chromosomes 16, 21, and 22 being the most prevalent autosomal chromosomes abnormalities. However, in the SMF subgroups with younger female partners, the occurrence of abnormalities for chromosomes 2, 11, 17, 21 and XY was also more frequent. This suggests that SMF increases the risk of aneuploidy whether or not female age is factored in. A significant increase in all these chromosomes was observed in NOA patients. The previous study by Coates et al. (2015) indicated that severe male infertility is associated with a significant increase in the occurrence of sex chromosomal abnormality in blastocyst embryos compared with embryos derived from normal semen samples [6]. Their findings regarding chromosomes 2, 11 and sex chromosomes were similar to ours. However, in addition, our study identified an increase in the rates of abnormality in chromosomes 10, 17 and 21. The abnormality of chromosome 21 has commonly been considered to be maternal in origin. Because our study focused on SMF cases with young female partners, the significant increase in the rate of chromosome 21 abnormalities suggests a link with SMF. Carrell et al. (2004) reported that chromosome 21 was one of five chromosomal abnormalities in male infertility cases observed in their FISH study. They concluded that the severity of sperm chromosome aneuploidy appears to be proportional to the severity of abnormal semen quality: in particular, abnormal morphology [28]. Wegetti et al. (2000) reported a positive correlation between semen parameters and sperm aneuploidy rates investigated by fluorescence in situ hybridization in infertile men and that, in the overall group of infertile men, there was a significantly increased frequency of disomy for chromosomes 13, 18, 21, XX, YY and XY [29].

In testicular sperm patients, significantly high incidence of sex chromosome abnormality was found. In both our study and in the literature, peripheric karyotype results have shown a similarly high incidence of sex chromosome abnormality in SMF cases. These findings suggest that the vast majority of chromosomal abnormalities in embryos in NOA cases are paternal in origin.

Both Hassold et al. (1996) and Griffin et al. (1996) reported that 50% of cases with 47XXY and 100% of cases with 47XYY were paternal in origin. Increases in aneuploid and diploid sperm lead to elevated rates of abnormal and mosaic embryos that may be potentially viable but also have sex chromosome aneuploidy [30, 31]. FISH studies on testicular sperm from azoospermic patients with normal karyotype confirm an increase of mainly sex chromosome abnormalities [32]. These sex chromosome aneuploidies could eventually lead to implantation failure and miscarriages.

However, it is important to note that, despite the increase in rates of abnormality, once a chromosomally normal embryo was found, no difference was observed in clinical outcomes in our SMF subgroup. This could be a consideration when patients with SMF are counseled.

Sperm play an essential role in embryonic genome activation. Abnormal sperm parameters and chromatin alteration affect the normal embryo kinetics in ICSI program. Several studies have examined the morphokinetic development of embryos in SMF cases [33–37, 7]. We, too, evaluated the morphokinetic development of embryos, but, in addition, investigated any possible correlation between morphokinetics and chromosomal status in SMF cases. In a time-lapse imaging study, aneuploid embryos showed a delayed initiation of blastocyst formation and reached the full blastocyst stage later compared with euploid embryos [38].

In SMF cases, defective centrosome plays an important role in the embryo development. Centrosome is paternally inherited and responsible for the first mitotic divisions after fertilization. The final stage of fertilization is mediated by the sperm centrosome, which induces microtubule organization into the first meiotic spindle and provides the precursor and most critical for all developmental stages from the fertilization to late stage development A defect in sperm aster formation could have serious consequences for later development, possibly resulting in multiple mitotic spindles, disorganized chromosomes, or improper cell divisions [39–42]. Paternally derived portion of the centrosomes varies among males and that this variation effect male fertility, the outcome of early embryonic development and therefore reproductive success. [43].

Comizzoli et al. (2006) showed a higher proportion of zygotes with short or absent sperm asters after ICSI with testicular spermatozoa compared with ejaculated spermatozoa that contained large sperm asters after ICSI [8]. The poor pattern of aster formation from the testicular centrosome was associated with delayed first cleavage, slower developmental rate and reduced formation of morulae and blastocyst. Remarkably, improvement was reported when testicular sperm centrosome was replaced by a centrosome from an ejaculated spermatozoon that resulted in higher rates of embryo development comparable with data from ejaculated spermatozoa.

Similarly, in our study, when time to first cleavage (t2) and blastocyst were evaluated, in normozoospermic cases, euploid embryos developed faster than aneuploid embryos. However, in the most severe forms of male infertility with testicular sperm and less than 1 mil/ml spermatozoa, there was no such difference between the euploid, aneuploid and mosaic embryos. These findings suggest that, in testicular sperm and < 1 mil/ml sperm groups, morphokinetic evaluation alone does not provide sufficient information about euploid, aneuploid and mosaic embryos. The correlation between morphokinetics and chromosomal status is greatly reduced or disappears in the most severe forms of male infertility. This indicates the need for new morphokinetic models for very severe forms of male infertility.

In conclusion, although many areas for further investigation remain—Why does testicular sperm differ chromosomally from ejaculated sperm? Why does the rate of mosaicism significantly increase in SMF? Why does the correlation between morphokinetic development and chromosomal status disappear in the most severe forms SMF?—our study observed high rates of aneuploidy, mosaicism and abnormal morphokinetic development in cases with testicular and < 1 mil/ml sperm groups with female partners ≤ 35 years. These findings suggest that PGT-A may be an indication in severe male infertility cases. Furthermore, the correlation between morphokinetics and chromosomal status was greatly reduced or disappeared in these most severe forms of male infertility, thus the need for new morphokinetic models.

## Electronic supplementary material

ESM 1 (DOCX 27 kb).

ESM 2 (DOCX 29 kb).

ESM 3 (DOCX 14 kb).

ESM 4 (DOCX 15 kb).
