# Supplementary material for: High rates of aneuploidy, mosaicism and abnormal morphokinetic development in cases with low sperm concentration
Source: J Assist Reprod Genet. 2020 Jan 4;37(3):629–40. doi: 10.1007/s10815-019-01673-w (PMC7125256; doi:10.1007/s10815-019-01673-w)
Supplement: Supplementary file 1 — (DOCX 27 kb). [file 10815_2019_1673_MOESM1_ESM.docx]

Supplementary Figure 1. Overall karyotype abnormalities in all SMF cases (n=3276) between 2003–2018. Ejaculated sperm cases <5mil/ml and Testicular sperm cases.
