# Supplementary material for: High rates of aneuploidy, mosaicism and abnormal morphokinetic development in cases with low sperm concentration
Source: J Assist Reprod Genet. 2020 Jan 4;37(3):629–40. doi: 10.1007/s10815-019-01673-w (PMC7125256; doi:10.1007/s10815-019-01673-w)
Supplement: Supplementary file 2 — (DOCX 29 kb). [file 10815_2019_1673_MOESM2_ESM.docx]

Supplementary Figure 2. Distribution of autosomal and sex chromosome abnormalities according to SMF, women age ≤35 (All PGT-A tested embryos: 1570 blastocysts (741 for the study group, 829 for the control group)).
