# Supplementary material for: High rates of aneuploidy, mosaicism and abnormal morphokinetic development in cases with low sperm concentration
Source: J Assist Reprod Genet. 2020 Jan 4;37(3):629–40. doi: 10.1007/s10815-019-01673-w (PMC7125256; doi:10.1007/s10815-019-01673-w)
Supplement: Supplementary file 3 — (DOCX 14 kb). [file 10815_2019_1673_MOESM3_ESM.docx]

Supplementary Table 1. Patients and cycle characteristics according to SMF subgroups with young female partners (≤35 years) (mean values and standard deviations are given).

|  | < 1 mil/ml | 1-5 mil/ml | Testicular sperm | Control Group |
| --- | --- | --- | --- | --- |
| No of cases | 128 | 115 | 36 | 165 |
| Number of Cycles | 137 | 150 | 39 | 190 |
| Female Age | 31.1 ± 3.5 | 31.1 ± 3.7 | 31.3 ± 3.9 | 30.8 ± 3.7 |
| Male Age  (Minimum - Maximum) | 34.7 ± 4.7  (22 – 45) | 33.9 ± 3.8  (25 – 43) | 33.3 ± 4.2  (22 – 44) | 32.8 ± 4.8  (23 – 47) |
| BMI | 23.6 ± 7.8 | 23.1 ± 7.2 | 23.4 ± 6.0 | 24.3 ± 7.1 |
| AMH | 2.9 ± 2.5 | 3.1 ± 2.8 | 2.9 ± 3.2 | 3.6 ± 2.2 |
| COC | 15.2 ± 3.6 | 15.7 ± 3.4 | 15.1 ± 3.0 | 16.4 ± 4.2 |
| MII oocytes | 13.2 ± 3.2 | 13.8 ± 2.9 | 13.4 ± 2.4 | 14.1 ± 3.8 |
| Fertilization % | 81% | 81% | 77.3% | 83% |
