# Supplementary material for: High rates of aneuploidy, mosaicism and abnormal morphokinetic development in cases with low sperm concentration
Source: J Assist Reprod Genet. 2020 Jan 4;37(3):629–40. doi: 10.1007/s10815-019-01673-w (PMC7125256; doi:10.1007/s10815-019-01673-w)
Supplement: Supplementary file 4 — (DOCX 15 kb). [file 10815_2019_1673_MOESM4_ESM.docx]

Supplementary Table 2. Morphokinetic evaluation of embryos in SMF groups, with young female partners (≤ 35 years).

|  | Testicular Sperm  (n=43) | <1 mil/ml  (n=81) | 1-5 mil/ml  (n=82) | Control Group  (n=421) | p* |
| --- | --- | --- | --- | --- | --- |
| t2 | 26.8  (25.4 to 27.9) | 26.8  (26.2 to 27.2) | 27.0  (26.4 to 27.8) | 27.0  (26.5 to 27.5) | 0.048 |
| t3 | 36.7  (35.3 to 39.1) | 37.1  (36.0 to 37.9) | 37.3  (36.6 to 37.9) | 37.4  (36.7 to 38.1) | 0.005 |
| t4 | 38.8  (37.2 to 40.2) | 38.8  (37.9 to 39.6) | 39.0  (38.2 to 39.8) | 39.1  (38.5 to 39.7) | 0.027 |
| t5 | 48.2  (46.5 to 50.4) | 49.3  (47.5 to 50.4) | 49.7  (48.1 to 51.5) | 49.8  (49.2 to 50.4) | <0.001 |
| t6 | 51.9  (49.5 to 53.9) | 52.0  (50.5 to 52.9) | 52.4  (51.4 to 53.5) | 52.6  (52.1 to 53.1) | <0.001 |
| t7 | 54.5  (52.6 to 56.2) | 54.0  (52.7 to 55.2) | 54.7  (53.4 to 55.7) | 55.1  (54.6 to 55.7) | <0.001 |
| t8 | 57.4  (55.2 to 60.4) | 57.3  (56.1 to 58.2) | 57.2  (56.0 to 58.8) | 57.8  (57.2 to 58.3) | 0.007 |
| t9 | 67.5  (65.1 to 69.6) | 68.5  (67.1 to 69.9) | 69.3  (68.1 to 70.6) | 69.3  (68.7 to 70.0) | <0.001 |
| tM | 86.9  (83.2 to 89.5) | 87.6  (85.7 to 89.9) | 85.9  (84.0 to 87.3) | 87.5  (86.8 to 88.2) | 0.021 |
| tSB | 96.2  (93.5 to 98.9) | 97.0  (95.1 to 98.8) | 97.2  (95.7 to 98.2) | 97.7  (97.6 to 98.3) | 0.042 |
| tB | 104.1  (100.4 to 108.1) | 105.5  (103.5 to 107.6) | 104.8  (102.2 to 107.2) | 105.9  (104.2 to 107.4) | 0.021 |

*Kruskal – Wallis Test, median values and 95% CI for median are given,
